# Thinning or Opening: A Randomized Sibling-Embryo Pilot Trial on the Efficacy of Two Laser-Assisted Hatching Modes During the Extended Culture of Highly Fragmented Cleavage Embryos

**DOI:** 10.3389/fendo.2022.927834

**Published:** 2022-06-27

**Authors:** Ling Zhang, Yi-er Zhou, Yue-jin Wu, Li-mei Wu, Shi-shi Li, Lin Zhang, Zhen Jin, Chong-yi Shu, Wei-hai Xu, Jing Shu

**Affiliations:** ^1^Reproductive Medicine Center, Department of Reproductive Endocrinology, Zhejiang Provincial People’s Hospital, Affiliated People’s Hospital, Hangzhou Medical College, Hangzhou, China; ^2^Institute of Food Science and Engineering, Hangzhou Medical College, Hangzhou, China

**Keywords:** blastocyst, fragmentation, laser-assisted hatching (LAH), *in vitro* fertilisation and embryo transfer, oocyte utilization

## Abstract

A randomized sibling-embryo pilot trial investigated whether two ways of laser-assisted hatching result in different blastulation and clinical outcomes after extended *in vitro* culture process of highly fragmented day-3 cleavage embryos. From 92 couples, a total of 315 highly fragmented day-3 embryos (the fragmentation >25%) were recruited and randomized into laser-assisted zona thinning (LAT, n=157) and opening (LAO, n=158) groups, and then underwent a blastocyst culture *in vitro*. The main endpoint measurements including blastocyst formation and grading as well as the clinical pregnancy after blastocyst transfer were obtained during the treatment procedure of *in vitro* fertilization and embryo transfer, and then analyzed with generalized estimating equation (GEE) and/or time-to blastocyst analysis models. A total of 166 day-3 embryos developed into blastocyst stage (52.70%), of which 97 were viable blastocysts (30.79%), and 42 top-quality ones (13.33%). LAT did not have any inferior or superior to LAO in the endpoints of either total, viable, top-quality or hatched blastocyst formation, with the ORs (95%CI) from GEE model as 0.89 (0.55-1.45), 0.71 (0.42-1.21), 1.12 (0.56-2.25) and 0.68 (0.42-1.12) respectively for LAT treatment. And the time-to-blastocyst analysis showed a similar result. Additionally, no difference in clinical outcomes after blastocyst transfer was found between the two groups. The author concluded that when applying the LAHs during the extended culture of highly fragmented embryos, both LAT and LAO can generate a promising clinical outcome, and the LAT operation be equivalent to the LAO. Future well-designed, multiple-center, larger-sample investigations are required to ascertain above conclusion.

## Introduction

Embryonic fragmentation, one of the most common morphological defections in cleavage embryos, is considered as a dominant impairment to oocyte utilization during IVF procedure, and unanimously as a major stubborn challenge faced by embryologists (1, 2). Despite poor developing potential and inappropriateness for direct transfer, heavily fragmented cleavage embryos are still subject to extended *in vitro* culture in practice, with the purpose of vitality discrimination (3, 4). Currently nevertheless, the blastulation of the fragmented embryos remains really inferior, even though various means have been attempted, such as mechanical aspiration of the cellular debris and addition of some cytokines such as granulocyte colony-stimulating factor (G-CSF) (5, 6). This highlights the necessity that additional modifications be considered for present blastocyst culture procedure

Laser technology is now being widely used in embryo laboratory operations such as intracytoplasmic sperm injection, embryo biopsy, sperm immobilization/selection, assisted hatching and others, and reaches a grate convenience and consistency (7). Laser-assisted hatching (LAH), which refers to etching the zona pellucida of embryos to facilitate the hatching process, has been regarded as the most frequent form of its applications (7, 8). A beneficial effect of LAH application on *in vitro* blastulation process has been implicated by abundant evidence that zona ablations by laser in cleavage embryos result in a better implantation potential and clinical pregnancy, especially for the cases of poor prognosis (9, 10). And the direct evidence related to this benefit has been reported in our and other’s studies (11, 12). All these findings encourage the addition of LAH operation during the extended culture process for untransferable cleavage embryos to promote the utilization of low-graded embryos, especially for the fragmented ones. Our previous report has identified an improved blastocyst outcome when a LAH operation was performed in low-graded cleavage embryos at day 4 (3). This is consistent with a previous study conducted on fragmented embryos using a AH method with acidic tyrode’s solution (13).

When performing LAH, two major types of zona ablation, zona thinning (LAT) and zona opening (LAO), are usually chosen (7, 14). Despite their clinical efficacy during ART treatment has been extensively studied and recognized, it remains elusive which one may bring about a better outcome (15). Some population studies showed that the two ways have a similar efficacy in the endpoints of implantation and clinical pregnancy (9, 16, 17), but others proposed that the former be associated with a better clinical outcome (18, 19). Evidence from animal studies show that LAO may result in a higher rate of hatched blastocysts than LAT (quarter zonal-thinning), while the blastocyst formation be similar (20, 21). Therefore, for a more promising prognosis, details especially the types of laser ablation should be explored furtherly when performing the LAH for the fragmented embryos. For this purpose, we conducted this prospective study with a randomized control sibling-embryo design to compare the clinical efficacy of the two ways of LAH during the extended culture of highly fragmented cleavage embryos.

## Materials and Methods

### Study Participants

This is a prospective randomized sibling-oocyte study on the IVF-ET couples from June, 2020 to April, 2021 at the Reproductive Center of Zhejiang Provincial People’s Hospital, China. The study protocol was approved by the Ethics Committee of Zhejiang Provincial People’s Hospital.

We recruited the participants from the infertile couples who underwent their first IVF-ET cycles. The inclusion criteria were defined as follows (1): female partner aged <40 years (2); those couples with more than 2 highly fragmented day-3 cleavage embryos (specified as embryos originating from 2PN zygote, with fragment rate >25% and at least 4 blastomeres) (3); receiving extended *in vitro* culture. The exclusion criteria were (1) abnormal karyotypes of any partner (2); embryos originating from assisted oocyte activating or *in vitro* maturation procedure (3); familial infertility of any partner. All recruited couples signed the written inform consents.

### Blastocyst Culture and Laser Operations

Controlled ovarian stimulation, oocyte retrieval and insemination were conducted according to the regular procedure of our routine clinical IVF-ET program. The zygotes formed were put into Sydney IVF Cleavage medium (CM, COOK MEDICAL, Australia) for further culture, and the forming embryos scored in light of the parameters of blastomere amount, blastomere evenness as well as fragment rate (22). The recruited day-3 fragmented embryos were arranged randomly into zona opening (LAO) or zona thinning (LAT) groups with a computer randomization method. Randomized embryos were put into blastocyst culture in parallel with the resting ones in 60 μL microdrops of Sydney IVF Blastocyst medium (Cook Medical, Australia) under routine conditions, 37°C, 6% CO_2_ and 5% O_2_.

At day 4, a laser dissection was performed for the two randomized groups as described previously (23). Briefly, in LAT group, a series of successive ablations of 2.6 ms duration, which covered quarter of the zona circumference, were performed at the outer edge of the zona to form a defect occupying about 60% zona thickness, while in LAO group, two juxtaposed rows of ablations with 2.6 ms duration were exerted perpendicular to the zona to create a single full thickness opening (about 10μm diameter) through the zona. Zona operation was carried out at the area that is farthest from the blastomeres both in the LAT and LAO groups with OCTAX Laser Shot system (MTG Medical Technology, Altdorf, Germany). At the morning day 5 and 6, the blastulation and hatching state was checked, and the quality of blastocysts were evaluated according to the Gardner score system (24). Specifically, those staged over 3 with inner cell mass or trophectoderm grade above ‘B’ were regarded as viable blastocysts, while those staged over 3 at day 5 or over 4 at day 6 and graded above ‘B’ for both inner cell mass and trophectoderm as good-quality ones. All viable and good-quality blastocysts were cryopreserved on day 5 or day 6 according to the instructions provided by the vitrification reagent manufacturer (Vitrolife, Sweden).

### Endometrial Preparation, Frozen Blastocyst Transfer and Pregnancy Check

Receptive endometrium was prepared through either a hormone replacement (HRT) or natural cycle. For HRT cycles, an oral dose of 6 mg/day estradiol (estradiol valerate, Delpharm Lille S.A.S, France) was given from cycle day 3 onwards, and if endometrial lining remained <7mm 7 days after HRT initiation, additional dose (5g/day) of transvaginal estradiol gel (Besins Manufacturing Belgium) was administrated for another 5-7 days. When endometrial thickness reached a satisfactory level, daily 40 mg progesterone was injected. For natural cycles, the progesterone treatment started from ovulation day or 2 days after LH surge. One or two blastocysts were thawed, and transferred under ultrasound guidance. Once pregnancy was achieved, the progesterone and estradiol supplementation were continued until 10 weeks of gestation.

Pregnancy was checked by serum hCG test 10 days after transfer, and ascertained under ultrasound examination 5 weeks after transfer. Clinical pregnancy referred to the presence of a gestational sac under ultrasound. An implantation rate was defined as the ratio of the number of gestational sacs visualized under ultrasound to that of embryos transferred, and early miscarriage rate as the spontaneous loss of a clinical pregnancy before 12th weeks of gestation.

### Statistics

The distribution of each continuous variable was checked by the Shapiro-Wilk test. If in normal distribution, the continuous variables were expressed as mean ± standard deviation, ( $\bar{x}$ ±SD) or else, d as median (inter-quartile Range, IQR). All category variables were described as n (%). Data analysis was performed with the SPSS statistical software (version 21.0, IBM Corp., USA). To compare the study endpoints and related covariates such as blastomere amount and fragments between the two groups, non-parameter Kruskal-Wallis test and Fisher’s exact test were conducted for continuous variables and category ones respectively. Importantly, a cluster-weighted generalized estimating equation (GEE) models were used to account for within person correlations between the fragmented embryos from the same participant, since the contribution of the cluster size (here refers to number of fragmented embryos) is nonignorable (25, 26). During GEE models, a binomial distribution with log link function were specified, and the inverse of the number of recruited embryos taken as the weighting coefficient. OR values were calculated after adjusting for the covariates of female age, BMI, primary infertility, intracytoplasmic sperm injection insemination (ICSI) as well as day-3 embryos parameters. Considering the fact that blastulation time be a determinant for further development and implantation potential, we also performed a time-to-blastocyst analysis by fitting conventional survival analysis, and assigned the blastocyst cryopreservation as the censoring event. A Cox proportional hazards regression model was specified for total and viable blastocyst formation, while a fine & Gray model for top-quality and hatched ones given the competitive relationship between cryopreserved blastocysts at day 5 and top-quality or hatched blastocysts formed at day 6. A two-tailed P<0.05 was considered as statistical significance.

## Results

### Recruited Couples and Embryos

A total of 92 couples and 315 fragmented day-3 embryos were recruited into present study. All participants were of Han ethnicity. The median age of female partners was 30 years [IQR: 28-32], average BMI 21.27 ± 2.59 kg/m^2^, median serum AMH level 4.49 [IQR: 2.85-7.14] ng/mL, and median retrieved oocytes 15 (IQR: 11-19). Other baseline characteristics of female partners were summarized in **Table 1**. Of all randomized day-3 embryos, the median number of blastomere was 5 [IQR:5-6], and median fragments 30% (IQR: 25%-35%). Finally, a total of 166 day-3 embryos developed into blastocyst stage (52.70%), of which 97 were viable blastocysts (30.79%), and 42 top-quality ones (13.33%). The blastulation outcomes here were obviously higher than those for untreated embryos as reported in our previous study (40.2%, 18.6% and 9.2%, respectively) under the same cultural conditions (3).

**Table 1:** Baseline characteristics of included couples.

Parameter	Values
No. of couples (n)	92
Female age (yrs.)	30 (28–32)
BMI (kg/m^2^)	21.27 ± 2.59
AMH (ng/mL)	4.49 (2.85-7.14)
Basic FSH (IU/L)	4.88 4.00-5.65)
AFC (n)	15 (10-25)
Infertility duration (yrs.)	2.0 (1.0-3.5)
Unexplained infertility [n (%)]	5 (5.43)
Primary infertility [n (%)]	46 (50.00%)
Controlled ovarian stimulating protocol [n (%)]	
Mini-stimulation	25 (27.17)
Long protocol	8 (8.70)
GnRH-ant	59 (64.13)
ICSI [n (%)]	38 (41.30)
Gn dose (IU)	2250 (1450-2700)
Days of Gn (d)	10 (9-11)
No. of oocytes (n)	15 (11-19)

Continuous variables were described as median (interquartile range). BMI, body mass index; AMH, anti-Mullerian hormone; AFC, antral follicle count; ICSI, Intracytoplasmic sperm injection.

### Blastocyst Cultural Outcomes

**Figure 1:**
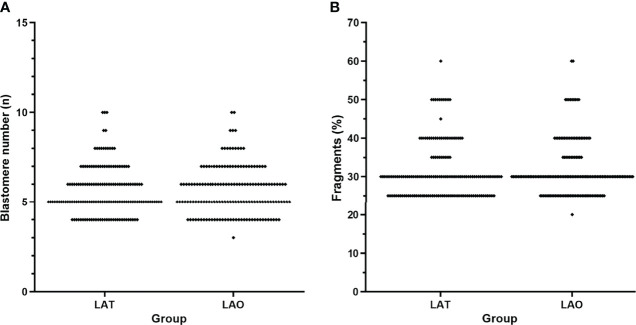
Distribution of blastomere number and fragments between the two groups. **(A)** blastomere number, and **(B)** embryo fragments. LAT, laser-assisted thinning; LAO, laser-assisted opening.

Of all recruited day-3 embryos, 157 were arranged to the LAT group, and 158 to the LAO. The distribution of blastomere amount and fragments were described in **Figure 1** and **Table 1**, and showed quite equivalent between the two groups, indicating a considerable comparability for the comparison. Consequently, total blastulation rates were 51.59% and 53.80%, viable blastocyst 28.03% and 33.54%, top-quality blastocyst 14.29% and 12.66%, and hatching blastocyst 31.85% and 31.24% for LAT and LAO treatments respectively. No significance between the two groups was observed in any above endpoints. At day 5, despite a seemingly lower viable and hatched blastocyst rate was identified in LAO group (17.20% vs 24.50%, and 14.65% and 22.78%), the difference did not reach a significance (**Table 2**).

**Table 2:** Characteristic and blastocyst cultural outcome between the two groups.

Parameter	LAT	LAO	P value*
n	157	158	
Blastomere amount at Day 3 (n)	5 (5-6)	5 (5-6)	0.972
Embryo fragment at Day 3 (%)	30 (25-35)	30 (25-35)	0.343
Blastocyst rate [n (%)]			
Total	81 (51.59)	85 (53.80)	0.695
Day 5	64 (40.76)	65 (41.14)	0.946
Viable blastocyst rate [n (%)]			
Total	44 (28.03)	53 (33.54)	0.289
Day 5	27 (17.20)	38 (24.05)	0.132
Top-quality blastocyst rate [n (%)]			
Total	22 (14.29)	20 (12.66)	0.724
Day 5	17 (10.83)	17 (10.76)	0.984
Hatched blastocyst rate [n (%)]			
Total	50 (31.85)	62 (39.24)	0.170
Day 5	23 (14.65)	36 (22.78)	0.061

*P value was calculated based by non-parameter Kruskal-Wallis test for continuous variable and Fisher exact test for categorical variables. LAT, laser-assisted thinning, LAO, laser-assisted opening.

Considering the risk that between-embryos correlation might impair the robustness of our study, a multi-variate analysis with cluster-weighted GEE (CWGEE) model were used to ascertain above results, and showed that difference in LAH ways have no influence on either total, viable, top-quality or hatched blastocyst formation, with the ORs (95%CI) from GEE model as 0.89 (0.55-1.45), 0.71 (0.42-1.21), 1.12 (0.56-2.25) and 0.68 (0.42-1.12) respectively for LAT treatment as compared with the LAO (**Table 3**). Tentatively, a time-to-blastocyst analysis grown out of survival analysis was conducted, and still showed a similar null result (ORs were shown in **Table 3**). Collectively, our results showed that zona thinning and opening have a similar blastulation outcome when used in fragmented day-3 embryos. Additionally, when analyzing the covariates taken into our multivariate models, we identified a striking association of blastomere amount and fragment rates with the blastulation outcomes in both CWGEE and time-to-blastocyst analysis (**Table 3**).

**Table 3:** GEE analysis and time-to-blastocyst analysis on the blastocyte culture outcomes of fragmented day-3 embryos.

Parameters		GEE model †				Time-to-blastocyst ‡		
	ORs*	95% CI		P values	ORs*	95% CI		P values
		Lower	Upper			Lower	Upper	
**Blastocyst formation**								
Female age	0.96	0.90	1.02	0.209	0.98	0.90	1.02	0.424
BMI	0.91	0.82	1.00	0.062	0.96	0.90	1.02	0.181
Primary infertility	0.61	0.36	1.03	0.064	0.81	0.90	1.02	0.214
Blastomere number	1.79	1.36	2.35	<0.001	1.24	0.90	1.02	<0.001
Fragment^#^	0.80	0.65	0.98	0.031	0.97	0.90	1.02	0.016
ICSI	1.52	0.90	2.56	0.117	1.13	0.98	1.11	0.441
LAH style	0.89	0.55	1.45	0.647	0.93	0.98	1.11	0.621
**Viable blastocyst formation**								
Female age	1.00	0.94	1.07	0.958	1.00	0.90	1.02	0.960
BMI	0.90	0.82	1.00	0.048	0.93	0.90	1.02	0.108
Primary infertility	0.72	0.42	1.24	0.235	0.81	0.90	1.02	0.339
Blastomere number	1.52	1.23	1.88	<0.001	1.30	0.90	1.02	<0.001
Fragment^#^	0.67	0.53	0.84	0.001	0.94	0.90	1.02	0.002
ICSI	1.03	0.60	1.77	0.926	0.98	0.98	1.11	0.927
LAH style	0.71	0.42	1.21	0.210	0.76	0.98	1.11	0.190
**Top-quality blastocyst formation**								
Female age	0.96	0.88	1.05	0.331	0.96	0.90	1.02	0.319
BMI	0.92	0.80	1.05	0.207	0.93	0.90	1.02	0.219
Primary infertility	1.32	0.63	2.76	0.456	1.27	0.90	1.02	0.426
Blastomere number	1.64	1.30	2.06	<0.001	1.47	0.90	1.02	<0.001
Fragment^#^	0.64	0.44	0.91	0.014	0.92	0.90	1.02	0.009
ICSI	0.57	0.27	1.23	0.154	0.64	0.98	1.11	0.151
LAH style	1.12	0.56	2.25	0.745	1.08	0.98	1.11	0.775
**Hatched blastocyst formation**								
Female age	0.99	0.93	1.06	0.856	0.99	0.90	1.02	0.740
BMI	0.89	0.80	0.98	0.022	0.92	0.90	1.02	0.012
Primary infertility	0.89	0.53	1.51	0.674	0.98	0.90	1.02	0.906
Blastomere number	1.57	1.26	1.94	<0.001	1.32	0.90	1.02	<0.001
Fragment^#^	0.97	0.93	1.01	0.103	0.97	0.90	1.02	0.036
ICSI	1.15	0.68	1.94	0.598	1.00	0.98	1.11	0.995
LAH style	0.68	0.42	1.12	0.132	0.73	0.98	1.11	0.063

^†^Cluster-weighted GEE analysis adjusted for the covariates of female age, BMI, primary infertility as well as day-3 embryos parameters;

^‡^For blastocyst and viable blastocyst formation, Cox proportional hazards regression model was used, and for top-quality and hatched blastocyst formation, the fine and Gray model used. Covariates were properly adjusted for the covariates of female age, BMI, primary infertility and ICSI as well as day-3 embryos parameters.

*When calculating the effect of LAT and primary infertility, LAO and secondary infertility were the references, respectively; Female age, BMI, blastomere amount and fragment were continuous variables here.

^#^The probability of total, viable, high-quality and hatched blastocysts with the amount of increasement of fragmentation rate by 5%.

### Clinical Outcomes After Blastocyst Transfer

To further compare the blastocyst development and implantation potential between the two groups, we also investigated the clinical outcomes after blastocyst transfer. Of all blastocysts derived from the recruited fragmented embryos, 29 were finally transferred, of which 16 were from the LAT group and 13 from the LAO. Clinical rates were 81.25% and 76.92%, live birth rates 62.50% and 69.23%, and miscarriage rates 23.08% and 10.00% for LAT and LAO groups respectively (**Table 4**). No significance was identified in any endpoints, either with univariate or GEE analysis models (**Tables 4**, **5**).

**Table 4:** Characteristics and clinical outcomes of blastocyte transfer.

Parameter	LAT	LAO	P values*
n	16	13	
Top-quality blastocyst [n (%)]	8 (50.00)	5 (38.46)	0.534
Clinical pregnancy [n (%)]	13 (81.25)	10 (76.92)	0.775
Ongoing pregnancy [n (%)]	10 (62.50)	9 (69.23)	0.704
Miscarriage [n (%)]	3 (23.08)	1 (10.00)	0.401

*P value was calculated based on Fisher exact test.

**Table 5:** Multi-variate analysis with GEE on clinical outcomes after blastocyst transfer.

	ORs*	95% CI		P values
		Lower	Upper	
Clinical pregnancy				
Female age	1.02	0.82	1.26	0.886
LAT	0.85	0.14	5.00	0.854
Ongoing pregnancy				
Female age	1.00	0.83	1.21	0.992
LAT	1.50	0.31	7.32	0.617
Miscarriage				
Female age	0.96	0.72	1.28	0.782
LAT	3.03	0.29	33.33	0.358

*Effect of LAT styles were evaluated as risk ratio (95% CI) with LAO operation as the reference.

## Discussion

Improving the utilization of highly fragmented embryos, the most common low-graded day-3 embryos, is conducive for the prognosis of current ART procedures. Our previous evidence has proven that LAH application may play certain beneficial role during the blastocyst culture of low-graded cleavage embryos, but which types of LAH may give a better outcome remains unknown. This randomized controlled study is the first to compare the blastulation and clinical outcomes of two types of LAH, quarter zona thinning (LAT) and single point opening (LAO) in the fragmented day-3 embryos. Our results showed that the LAT treatment have an equivalent value to LAO in terms of total, viable, top-quality and hatched blastocyst formation, as well as the clinical outcomes after blastocyst transfer. Both LAO and LAT have better blastulation outcomes compared to those of non-treated low-graded embryos, as reported in our previous study (3).

Fragments in early human embryos are generally considered as anucleate cytoplasmic structures, which usually occur during the cytokinetic phase of blastomeres (27, 28). General consensuses have been already reached on the poor prognosis of the fragmented embryos (particularly when the fragmentation >25%) (1, 4, 29, 30). For the cost-effective consideration, an extended culture is usually recommended to screen out the pitifully few survivals which have an acceptable developmental potential. In this study, we chose day 4 instead of day 3 to operate LAHs, because a larger perivitelline space can be obtained in day-4 compact embryos, which facilitates the operation and reduces the risk of damage to embryos. This prolonged process also provides a window period for certain interventions intended for a better blastulation, such as aspiration of the fragments, addition of some cytokines as well as LAHs. As shown in present and our previous study, application of LAHs in low-graded or highly fragmented cleavage embryos are related to an appreciable blastulation outcome (transferable blastocyst >30%), substantially better than that in the control (3).

Some mechanisms have been supposed for the harmful effect of embryo fragments. Deleterious microenvironment related to the fragments may cause a constant and permanent damage to the neighboring blastomeres. It is believed that fragments within embryos release some toxic substances through certain pathophysiological processes such as necrosis and apoptosis (31–33), which construct a deleterious microenvironment containing high reactive oxygen species and proapoptotic factors such as caspase-3 (32, 34). Ultrastructural observation showed that the blastomere adjacent to fragments present signs of degeneration, suggesting induced apoptosis indeed occur in peripheral cells (35). On the other hand, communication between blastomeres, mainly mediated by gap junction, has been recognized as the key regulator during blastomere radial polarization and compaction, and disturbance to this cellular event may impair the foundation for the initial cell fate decisions and morphogenetic movements of embryogenesis (36, 37). In highly fragmented embryos, the fragments occupying the space among blastomeres, may hamper that relationship, and consequently, impair morula formation, embryo cavitation and blastocyst formation (38). From this perspective, it seems that LAO might be superior to LAT when applied during extended culture of fragmented embryos, since an opening hole by LAO may promote the efflux of localized harmful substances such as ROS, proapoptotic factors, and even the fragment itself. Furthermore, an artificial opening through zona may facilitate the exchange of nutrients and other bioactive substances, which is requisite for embryo development and blastulation (3). Nevertheless, in present study, we found an equivalent beneficial effect for the two treatments. Similarly, despite some retrospective studies showed that the defragmentation operation on highly fragmented embryos may result in an equivalent clinical outcome to that of high-grade, non-defragmented embryos (2, 39), a randomized controlled study ascertained that fragment removal operation cannot increase the embryo’s ability to undergo compaction, morula formation, or blastocyst formation (5). These findings suggested that alleviating local deleterious environment or reducing the existence of debris alone have no, or minimal (if any) contribution the beneficial effect of LAHs in fragmented embryos.

Another likely explanation for the advantage of LAHs relies on advanced escape from encapsule of zona and shorter coexistence of the developing embryos with the adverse environments. Indeed, a bulk of evidence have proven that both LAO and LAT be able to accelerate the process of hatching, and some considered that these methods can increase the blastocyst formation (11, 12, 14, 40). In view of this, relieving the developing embryos from a deleterious circumstance caused by fragmentation might be the common pathway for the two LAHs, and explain the equal beneficial effect for LAO and LAT. Lastly, even though LAT cannot render additional benefits from thorough opening through the zona, it presents some unique advantages. There is less risk of blastomere damage with zona thinning due to the laser beam being positioned further away from the cells than LAO. Additionally, LAT can retain the remnant opportunity of blastocyst expansion and physiological zona thinning during extended culture, which has been supposed to boost the proliferation of blastocyst cells and then generate a blastocyst with putative higher grade (14, 20). In present study, we thinned outer layer of zona in the LAT group consistent with many other researchers (14, 40), retaining the more elastic inner layer. Taken together, the similarity in the blastulation outcomes between the two groups should be attributed to the comprehensive consequence of multiple mechanisms.

A multivariate weighted GEE model, which has been thought to be competent in handling the data of cluster type (25, 26), was adopt in present study to adjust for the confounding from certain covariates including female age, BMI, primary infertility, blastomere amount and fragment rate and ICSI operation, and meanwhile, control the close correlation within embryos from the same patient. Furthermore, the time-to-blastocyst endpoint were also analyzed with a Cox proportional hazards regression or fine and Gray model, which has been the first application of this statical method in the ART scene (41, 42). Both the cluster-weighted GEE and time-to-blastocyst analysis showed a consistent conclusion with that of univariate analysis, indicating a considerable robustness of our result. Additionally, when analyzing the covariates with the multivariate statistical models, a substantial contribution from both blastomere amount and fragments rates has been identified. The probability of total, viable, high-quality and hatched blastocysts increased by 79%, 52%, 64% and 57% respectively with the blastomere amount increased by 1, while decreased by 20%, 33%, 36% and 3% respectively with a 5% increasement in fragmentation. This finding may be of great implication for the future studies intended to estimate the difference in developing potential of fragmented embryos.

In summary, this study proposed that both LAT and LAO have an evident beneficial effect during the extended culture of highly fragmented day-3 cleavage embryos, and LAT be not inferior to LAO during this procedure. However, due to the strict inclusion criteria as well as priority of embryo selection for transfer in practice, even fewer recruited patients accomplishing a transfer with the blastocyst harvested in this study, the sample sizes for both cultural and transfer cycles remained even small, which might impair the statistical force. Thus, the conclusion derived from this study remains preliminary and should be treated with caution. Future well-designed, multiple-center, larger-sample investigations are required to provide a more solid conclusion.

## Data Availability Statement

The original contributions presented in the study are included in the article/supplementary material. Further inquiries can be directed to the corresponding authors.

## Ethics Statement

The studies involving human participants were reviewed and approved by Ethics Committee of Zhejiang Provincial People’s Hospital. The patients/participants provided their written informed consent to participate in this study.

## Author Contributions

LingZ and Y-EZ conducted the cultural manipulation, statistical analysis, and manuscript drafting. Y-JW, L-MW, S-SL, LinZ, ZJ, and C-YS contributed to data collection and critical discussion. W-HX and JS designed the study and edited the manuscript. The authors read and approved the final manuscript.

## Funding

This study was supported by the General Research Program for Medicine and Health of Zhejiang Province (2021KY065, LZ), ‘New Century 151 Talent Program’ of Zhejiang Province (LZ) and Adjunct Talent Fund of Zhejiang Provincial People’s Hospital.

## Conflict of Interest

The authors declare that the research was conducted in the absence of any commercial or financial relationships that could be construed as a potential conflict of interest.

## Publisher’s Note

All claims expressed in this article are solely those of the authors and do not necessarily represent those of their affiliated organizations, or those of the publisher, the editors and the reviewers. Any product that may be evaluated in this article, or claim that may be made by its manufacturer, is not guaranteed or endorsed by the publisher.
